# Three Concurrent Nonpalpable Nexplanon Implants in the Upper Arms Following Presumed Failed Insertions: A Case Report

**DOI:** 10.1155/crog/2797689

**Published:** 2026-09-17

**Authors:** Alexia Barrios, Kyara M. Marquez, Austin Zanelotti, Ian J. Bishop

**Affiliations:** ^1^ Tamer Family Department of Obstetrics, Gynecology, and Reproductive Sciences, University of Miami Miller School of Medicine, Miami, Florida, USA, miami.edu; ^2^ Department of Obstetrics and Gynecology, Baylor College of Medicine, Houston, Texas, USA, bcm.edu

**Keywords:** contraceptive implant, imaging studies, localization, Nexplanon, nonpalpable contraceptive implant, ultrasound

## Abstract

**Introduction:**

Subdermal etonogestrel implants (Nexplanon) are a widely used form of long‐acting reversible contraception, providing up to 5 years of pregnancy prevention. Their ease of outpatient insertion and high efficacy have contributed to their widespread use. However, correct subdermal placement and immediate postinsertion confirmation of palpability are essential to ensure proper positioning and facilitate future localization and removal.

**Case Presentation:**

We report the case of a 42‐year‐old G1P1001 patient who was ultimately found to have three nonpalpable concurrent Nexplanon implants in both upper arms following multiple presumed failed insertion attempts over several years. On several occasions, newly placed implants were not palpable after insertion and were assumed to represent failed deployment, leading to repeat insertions without imaging confirmation. At presentation to our institution, high‐frequency ultrasound identified a total of three nonpalpable, but superfascial, implants: two in the left upper arm and one in the right upper arm. All devices were successfully localized and removed in an outpatient setting using ultrasound‐guided techniques without complication.

**Discussion:**

Nonpalpable contraceptive implants most commonly result from deep insertion into subcutaneous, fascial, or intramuscular tissue. Most published reports describe single retained implants managed with high‐frequency ultrasound as the first‐line modality for localization and removal. This case is unique in demonstrating the cumulative consequence of repeated misinterpretation of nonpalpability as failed insertion, resulting in multiple retained devices across both upper arms. It highlights a critical gap in procedural verification and reinforces the importance of standardized postinsertion confirmation protocols.

## 1. Introduction

The progestin‐only, subdermal contraceptive implant (Nexplanon) is a popular form of long‐acting, reversible contraception. It is approved by Food and Drug Administration (FDA) for up to 5 years use and insertion/removal procedures are designed to be safely performed in an outpatient setting with local anesthetic. The precise insertion site is approximately 8–10 cm (3–4 in.) from the medial epicondyle of the humerus and 3–5 cm (1.25–2 in.) posterior to (below) the sulcus (groove) between the biceps and triceps muscles [1]. This location is specifically chosen to avoid the large blood vessels and nerves lying within and surrounding the sulcus—most notably the brachial artery, basilic vein, and median nerve, which run in the medial bicipital groove. Placement in the nondominant arm reduces discomfort and functional limitation during the postinsertion healing period, as the dominant arm is preserved for daily activities.

Correct insertion technique is essential to ensure subdermal placement and palpability. The inner (medial) aspect of the upper arm provides a relatively protected, soft‐tissue area where the implant can be placed subdermal and remain palpable—which is critical for confirming correct placement and facilitating future removal. Failure to achieve superficial placement may result in deep insertion, rendering the implant nonpalpable and complicating localization and removal. In such cases, imaging modalities such as radiography or high‐frequency ultrasonography are recommended to confirm the presence and position of the device.

We present a case of three concurrent nonpalpable Nexplanon implants in both upper arms, resulting from repeated insertions. Rather than using imaging modalities to identify deep placement, it was assumed the implants were not deployed (failed to insert).

## 2. Case Report

A 42‐year‐old gravida 1 para 1 (G1P1001) female with amenorrhea since her initial Nexplanon insertion in 2016 and no significant past medical history was referred to our clinic for evaluation and removal of multiple nonpalpable contraceptive implants.

A detailed history revealed that implants had been placed at multiple visits by different providers between 2016 and 2023. In 2016, the patient underwent uncomplicated insertion of a Nexplanon implant in the left upper arm by her primary care provider. In 2020, she returned for removal and reinsertion. Although the removal was uncomplicated, the newly placed implant was nonpalpable, and the insertion was presumed to have failed due to device malfunction. A subsequent attempt was made in the right upper arm at a follow‐up visit; however, this implant was also nonpalpable, and no imaging was performed. It was again assumed that insertion had not occurred and she was incorrectly informed that insertions had been unsuccessful.

A subsequent provider the same year, unaware of the previously retained nonpalpable Nexplanon devices, performed an additional uncomplicated implant insertion in the left upper arm, resulting in the presence of multiple implants. In 2023, she returned for removal and reinsertion. Although removal was uneventful, the newly placed implant was nonpalpable. An x‐ray performed at that time demonstrated two implants in the left upper arm, prompting referral to our clinic for further evaluation and management.

**Figure 1:**
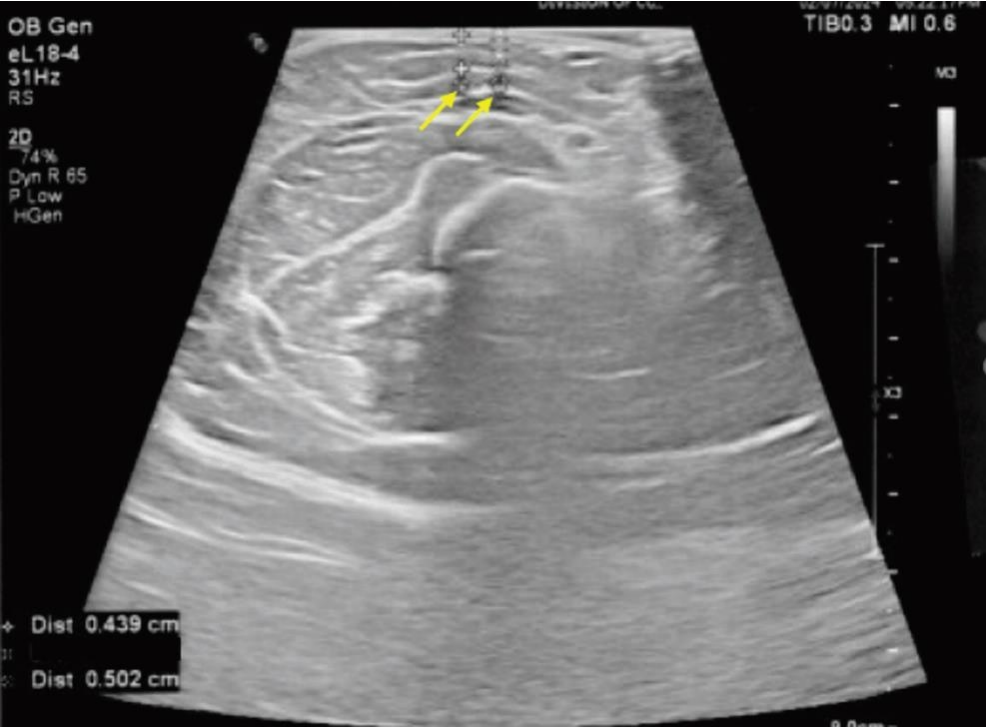
Ultrasound image obtained during evaluation of the upper left extremity. A high‐frequency transducer was used to confirm the presence of two nonpalpable Nexplanon implants (yellow arrows) located suprafascial above the muscle within subcutaneous tissue.

Given the clinical history, the presence of three implants across both upper arms was suspected. The left arm demonstrated multiple prior insertion and removal sites without palpable devices. The right arm had a single insertion site without a palpable device. High‐frequency ultrasound (≥ 10‐MHz linear transducer) localized two implants in the left upper arm within the subcutaneous tissue, positioned suprafascially at depths of 4.39 and 5.02 mm from the skin surface (Figure 1). The implant in the left arm was 3.00 mm from the skin surface.

The patient was counseled on management options and elected to undergo in‐office ultrasound‐guided removal. Each implant was localized with the ultrasound and the proximal and distal ends marked on the skin. After sterile preparation, local anesthesia was achieved with 2 cc of 1% lidocaine with epinephrine midimplant. A small longitudinal incision (approximately 3–5 mm) was made midimplant, blunt dissection was used through subcutaneous tissue down the implant with a mosquito hemostat under ultrasound guidance, and the implant was grasped with a modified vasectomy clamp encircling and securing the rod. The implant was gently raised to the surface, the surrounding scar capsule was taken down with a scalpel, and removed intact. This process was repeated for all three implants. Hemostasis was achieved, and the incision sites were closed with adhesive strips and sterile dressings. All implants were successfully removed without complication.

Despite prolonged exposure to multiple implants, the patient reported no adverse effects commonly associated with progestin‐containing contraceptives, including abnormal bleeding, mood changes, or weight gain.

She was counseled on alternative contraceptive options and initiated a progestin‐only pill.

## 3. Discussion

Nonpalpable etonogestrel implants are a well‐described complication of subdermal contraceptive use, most attributed to deep insertion within the subcutaneous tissue, fascial plane, or underlying muscle rather than true device translocation [2]. Published case reports and series consistently describe diagnostic and management delays when nonpalpability is initially misinterpreted as failed insertion, underscoring the importance of early confirmation of device location [3–5].

In most previously reported cases, a single nonpalpable implant prompts imaging evaluation—typically with high‐frequency ultrasound as first‐line, followed by radiography due to the radiopaque nature of Nexplanon [6]. A high‐frequency linear probe (commonly used for vascular access) can be placed transversely (short axis) across the expected implant location. The implant appears as a bright, hyperechoic linear structure (echogenic focus) with posterior acoustic shadowing. Scanning in short axis first helps identify the implant cross‐section, then rotating to long axis confirms its full length and orientation. These approaches reliably localize the device and guide either office‐based or operative removal. Singh et al. [7] described a series of 27 patients with nonpalpable Implanon devices in whom ultrasound successfully localized nearly all implants and facilitated safe removal. More recent reports have demonstrated successful minimally invasive ultrasound‐guided extraction of deeply located implants, including subfascial and intramuscular devices situated near neurovascular structures [8].

In contrast to these previously described scenarios, the present case is distinguished not by difficulty in localization or removal but by the failure of recognition across multiple clinical encounters, resulting in the sequential insertion of three separate implants. This represents a distinct system‐level and cognitive error pattern that is not well described in the literature. Rather than a single retained device requiring localization, this patient experienced repeated reinforcement of an incorrect assumption—namely, that nonpalpability equated to nondeployment.

This distinction is clinically important. In prior reports, once a nonpalpable implant is identified, a diagnostic pathway is typically triggered, including imaging confirmation and avoidance of further insertion until resolution. In this case, that pathway was never initiated. Instead, the absence of palpability was interpreted as procedural failure, leading to repeat insertion attempts and accumulation of devices. This represents a breakdown in adherence to established insertion standards, which explicitly recommend immediate postinsertion palpation by both clinician and patient as the primary method of confirmation, with imaging reserved for any uncertainty [9].

The management of nonpalpable implants in the literature also highlights an important contrast with our case. Published reports emphasize escalation from office‐based ultrasound localization to specialist referral when necessary, but always within a framework of device confirmation prior to reinsertion. Guiahi et al. [10] described removal of an implant embedded within the biceps muscle using combined ultrasound and fluoroscopic guidance after failed office removal attempts. Similarly, referral‐center case series have demonstrated that most nonpalpable implants can ultimately be removed successfully in the outpatient setting using point‐of‐care ultrasonography and minimally invasive techniques [11]. Our case diverges from this paradigm, demonstrating how failure at the earliest step—confirmation of presence—can override later safeguards such as imaging availability and procedural expertise.

From a technical perspective, the successful ultrasound‐guided removal of all three implants in this patient aligns with prior literature demonstrating high success rates for minimally invasive retrieval of nonpalpable Nexplanon devices when they are localized within the subcutaneous or subfascial plane [12]. The depths observed in this case (4.3 and 5.0 mm) are consistent with prior reports of suprafascial misplacement, which are typically amenable to outpatient removal without operative intervention. However, the presence of multiple devices in different anatomic locations required sequential localization and extraction, adding complexity not commonly described in prior case reports.

Importantly, this case also contributes to the limited literature on clinical outcomes associated with prolonged and repeated etonogestrel exposure. Despite the presence of multiple implants over several years, the patient did not experience significant adverse systemic effects. Although pharmacokinetic studies have shown that serum etonogestrel levels may remain within contraceptive thresholds beyond the currently approved duration of use, there are minimal published data describing prolonged simultaneous exposure from multiple implants [13]. This case therefore adds clinically relevant observational data regarding tolerability in the setting of cumulative exposure, although broader conclusions cannot be drawn from a single patient.

Overall, this case illustrates that the principal failure was not technical difficulty in insertion or removal, but rather a breakdown in diagnostic reasoning—specifically, failure to consider nonpalpability as a marker of possible deep placement requiring confirmation. Existing literature has focused primarily on procedural management of nonpalpable implants; our case expands upon this by emphasizing how cognitive and systems‐level errors may contribute to repeated unnecessary insertions and delayed recognition of retained devices.

## 4. Conclusion

Nonpalpable Nexplanon implants should be presumed to represent possible deep or malpositioned devices until proven otherwise. This case highlights a rare but important consequence of misinterpreting nonpalpability as failed insertion, leading to repeated insertions and accumulation of multiple retained implants across different anatomic sites.

In contrast to previously published reports that primarily focus on localization and retrieval of single nonpalpable devices, this case underscores the downstream effects of failing to confirm device presence before reinsertion. It reinforces that immediate postinsertion palpation is a critical safety step and that imaging should be obtained prior to any repeat attempt when uncertainty exists.

Finally, this case demonstrates that ultrasound‐guided removal remains an effective and minimally invasive approach even in complex scenarios involving multiple retained implants, provided accurate preprocedural localization is achieved.

## Author Contributions

All authors have read and approved the final version of the manuscript. Ian J. Bishop, MD, MPH, had full access to all of the data in this study and takes complete responsibility for the integrity of the data and the accuracy of the data analysis. Ian J. Bishop, MD, MPH, affirms that this manuscript is an honest, accurate, and transparent account of the study being reported; that no important aspects of the study have been omitted; and that any discrepancies from the study as planned (and, if relevant, registered) have been explained.

## Funding

No funding was received for this manuscript.

## Ethics Statement

The authors have nothing to report.

## Conflicts of Interest

The authors declare no conflicts of interest.

## Data Availability

The data that support the findings of this study are available from the corresponding author upon reasonable request.
